# Sleep quality after stroke: A systematic review and meta-analysis

**DOI:** 10.1097/MD.0000000000033777

**Published:** 2023-05-17

**Authors:** Ye Luo, Guofeng Yu, Yuanfei Liu, Chengjun Zhuge, Yinge Zhu

**Affiliations:** a Quzhou College of Technology, Quzhou, China; b Department of Brain Surgery, People’s Hospital of Quzhou, Quzhou, China; c Department of Nursing, The Second Affiliated Hospital Zhejiang University School of Medicine, Hangzhou, China; d Affiliated Zhongshan Hospital Fudan University, Shanghai, China.

**Keywords:** meta-analysis, PSQI, sleep quality, stroke

## Abstract

Sleep-related problems are debilitating and long-lasting conditions in individuals with stroke. We aimed to estimate the prevalence of poor sleep quality after stroke by conducting a systematic review and meta-analysis.

**Methods:**

Five databases (PubMed, Embase, Web of Science, Scopus, and CINHAL) were searched for literature published before November 2022. Studies recruiting participants with stroke, using a validated scale to measure sleep quality and in English were included. We used the Agency for Healthcare Research and Quality Scale and Newcastle–Ottawa Scale to assess the quality of eligible studies. Pooled prevalence and subgroup analyses were performed to understand the variation in sleep quality among studies. We followed the PRISMA checklist to report the study.

**Results:**

Thirteen studies were included for analysis (n = 3886). The pooled prevalence of poor sleep quality was 53% (95% CI 41–65%). Studies using PSQI with a cutoff point of 7 had a prevalence of 49% (95% CI 26–71%), whereas those with a cutoff point of 5 had a higher prevalence of 66% (95% CI 63–69%) (*P* = .13). Geographical location could explain the prevalence variation between studies. The majority of included studies had a medium level quality of evidence (10/13).

**Conclusion:**

Poor sleep quality appears to be common in patients with stroke. Considering its negative impact on health, effective measures should be taken to improve their quality of sleep. Longitudinal studies should be conducted to examine the contributing factors and investigate the mechanisms that lead to poor sleep quality.

## 1. Introduction

Stroke is a medical condition in which poor blood flow to the brain causes cell death. According to the Institute for Health Metrics and Evaluation, stroke has become the leading cause of death and disability globally, which causes 11.59% of total deaths and 5.65% of total disability adjusted life years.^[1]^ A nationally representative survey in China showed that the prevalence of stroke was 1596.0/100,000 people in 2013, which was statistically greater than those reported 3 decades ago, especially among rural residents.^[2]^ Data from a global burden of disease study reported that there were 1.12 million incident strokes in the European Union in 2017 and 7.06 million disability-adjusted life years lost because of stroke. The number of people living with stroke is estimated to increase by 27% from 2017 to 2047.^[3]^ Despite heterogeneity in involved participants, the global epidemic of stroke has posed a great threat on population health over decades.

People living with stroke often have various complications and complex brain symptoms such as cognitive impairment, memory loss, dementia, fatigue and insomnia.^[4–6]^ Previous research has shown that sleep-related problems are common in patients with stroke.^[7,8]^ Stroke may lead to damage to the central nervous system and impair brain activity and sleep architecture since most of the anatomical structures of sleep are located in the central nervous system.^[6]^ All sleep disorders are associated with poor sleep quality, which consequently reduces patients’ quality of life and hinders their rehabilitation. However, there is a noticeable variability in the range of patients with poor sleep quality after stroke. This variation may be due to multiple reasons such as the methodological, geographical and clinical differences between different studies. The Pittsburgh Sleep Quality Index (PSQI) is a well-acknowledged scale to measure sleep quality over a 1-month time interval.^[9]^ Despite using the same scale, the difference in cutoff point may cause a considerable variation between estimates. Moreover, the time of assessment is likely to affect the prevalence of poor sleep quality as well. Therefore, there is still a lack of comprehensive, systematic evidence for the sleep quality among patients with stroke.

To address this gap, the objective of this systematic review was to estimate the prevalence of poor sleep quality after stroke and determine the factors of its variability. The evidence synthesis would have multiple implications for stroke survivors and their families’ lives.

## 2. Methods

### 2.1. Study selection

Electronic databases including PubMed, Embase, Web of Science, Scopus, and CINHAL (through EBSCO platform) were searched using key words related to “stroke” and “sleep quality” in title and abstract of the studies published from database inception to November 2022. Two reviewers (Y.L. and G.Y.) independently screened the titles and abstracts of each article and then reviewed the full-text to evaluate potentially relevant studies. Any differences regarding selection were settled via discussion with a senior author. The inclusion criteria were studies which: recruited adults with stroke; measured sleep quality using a validated scale at any time after stroke; were published in a peer-reviewed journal in English. Intervention studies were included if baseline or pre-intervention data of sleep quality were available. We excluded the studies which: were reviews, meta-analyses, letters, comments and conference abstracts; specified presence of sleep quality as an inclusion criterion; measured sleep quality using a single question (e.g., “How do you feel about your sleep quality?”). As for studies which reported data from the same cohort, we only included the study with the largest sample size. We also manually examined the reference lists of all included articles to identify relevant studies on the topic. Preferred Reporting Items for Systematic Reviews and Meta-Analyses was used to report this study.

### 2.2. Data extraction

Two reviewers independently extracted the data of each eligible study using a standardized form including first author, publication year, geographic location, study design, sample size, stroke type participants’ age, gender proportion, measurements of sleep quality, cutoff score, time of assessment after stroke and prevalence of poor sleep quality. Any further data or clarifications needed were requested directly from the authors via emails. Any differences regarding data extraction were settled via discussion with a senior author.

### 2.3. Quality assessment

To appraise the quality of included studies, we adopted the Agency for Healthcare Research and Quality Scale with a total score of 11 points to assess the quality of eligible cross-sectional studies. An item was scored as “0” with the answer for “no” or “unclear,” else as “1” for “yes.” We assigned scores of 0 to 3, 4 to 7, and 8 to 11 for low, moderate, and high quality, respectively.^[10]^ The quality of eligible prospective cohort and case–control studies was assessed using the Newcastle-Ottawa Scale including selection of participants, comparability of participants and assessment of outcomes, with a total score of 9 points. We assigned scores of 0 to 3, 4 to 6, and 7 to 9 for low, moderate, and high quality, respectively.^[11]^ Studies with poor quality were excluded. Two independent reviewers were involved in this process and any differences were settled via discussion with a senior author. In subgroup analysis, the quality of studies was seen as one of the potential sources of heterogeneity in the pooled analysis.

### 2.4. Statistical analysis

Meta-analysis was conducted using Review Manager 5.4 software. Studies that used the same measurement tool and cutoff score were pooled together. Prevalence data were recorded, and standard errors (SE) were computed using prevalence and the sample size from each study. The formula is $SE=\sqrt{p*\left(1-p\right)/n}$. Random effects model with the generic inverse variance method was performed to determine the pooled prevalence of poor sleep quality and heterogeneity between studies as it assumes that different factors affect the data variability other than a mere error or chance. Subgroup analyses were performed to compare prevalence across studies based on different factors such as location, stroke type and time point of assessment. A funnel plot was produced to assess the risk of publication bias.

### 2.5. Ethical review

Ethical approval was not necessary as this study was a systematic review and meta-analysis, and did not involve patients.

## 3. Result

### 3.1. Characteristics of included studies

**Figure 1.**
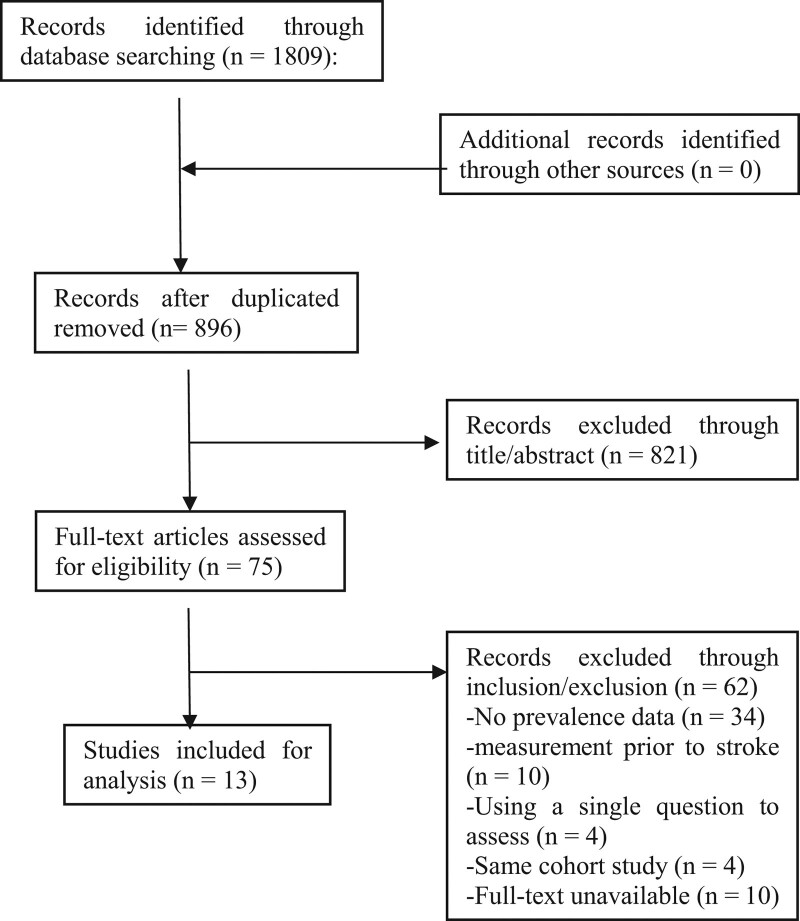
PRISMA flow diagram.

The initial search yielded 1809 articles. After removing duplicates, 896 studies remained. No additional paper was identified through other sources. Following a detailed screening of titles, abstracts and full-text, 13 studies (n = 3886) were selected for final analysis (Fig. 1). Of these papers, 5 were conducted in China, 2 in Brazil, and 1 in Vietnam, Japan, Cyprus, Iran, USA, and Hong Kong, respectively. There are cross-sectional studies and prospective studies in the included papers. The majority (n = 12) employed PSQI as measurement tool for sleep quality, except for one study using a self-developed questionnaire. A total of 6 studies adopted 5 as cutoff score while 5 studies regarded it at 7 and one set the cutoff score as 6. Characteristics of these 13 studies are outlined in Table 1.

**Table 1:** Characteristics of included studies (n = 13).

Study	Study design	N	Stroke type	Age	Male%	Measurement tool	Cutoff score	Time after stroke	Prevalence of poor sleep quality
Ho et al,^[12]^ Hong Kong	Cross-sectional study	112	NR	64.18 ± 5.77	66.1	PSQI	7	Mean 6.08 yr ± 4.80	64.3
Khazaei et al,^[13]^ Iran	Cross-sectional study	97	NR	67 ± 79	55.7	PSQI	7	≥1 mo	84
Zhang et al,^[14]^ China	Prospective cohort study	223	Ischemic	38.26 ± 6.35	76.2	PSQI	7	Upon admission	47.1
Sonmez and Karasel,^[15]^ Cyprus	Cross-sectional study	55	Ischemic (83.6%) Hemorrhagic (16.4%)	69 ± 11	50.9	PSQI	5	Upon admission	58.2
Pereira et al,^[16]^ Brazil	Cross-sectional study	43	Ischemic (67.4%) Hemorrhagic (32.6%)	56.9 ± 14.97	58.1	PSQI	5	Mean 6.1 ± 4.26	65.1
Xiao et al,^[17]^ China	Prospective cohort study	327	Ischemic	61.28	63.3	PSQI	7	1 mo	23.2
Kasai et al,^[18]^ Japan	cross-sectional study	51	Ischemic (59%) Hemorrhagic (41%)	72 ± 19	54.9	Self-developed questionnaire	/	1–3 mo	21.6
He et al,^[19]^ China	Prospective cohort study	191	Ischemic	61.50 ± 10.34	65.4	PSQI	7	2 mo	24.6
Zhao et al,^[20]^ China	Cross-sectional study	238	Ischemic (85.7%) Hemorrhagic (14.3%)	M = 61, P25 = 53, P75 = 68	68.1	PSQI	5	Upon admission	64.7
Fan et al,^[21]^ China	Prospective cohort study	1756	Ischemic	56.09	71.2	PSQI	5	3 mo	64.7
Del Brutto et al,^[22]^ USA	Prospective cohort study	561	NR	60.4 ± 12.6	42	PSQI	6	5 yr	30
de Oliveira et al,^[23]^ Brazil	Cross-sectional study	75	Ischemic and hemorrhagic (NR)	59.8 ± 12.9	51.5	PSQI	5	Upon admission	70.6
Nguyen et al,^[24]^ Vietnam	Cross-sectional study	157	Ischemic (64%) Hemorrhagic (36%)	73.1 ± 9.6	52	PSQI	5	1 yr	72

M = median, P25 = lower quartile, P75 = upper quartile, PSQI = Pittsburgh Sleep Quality Index.

### 3.2. Quality of included studies

**Figure 2.**
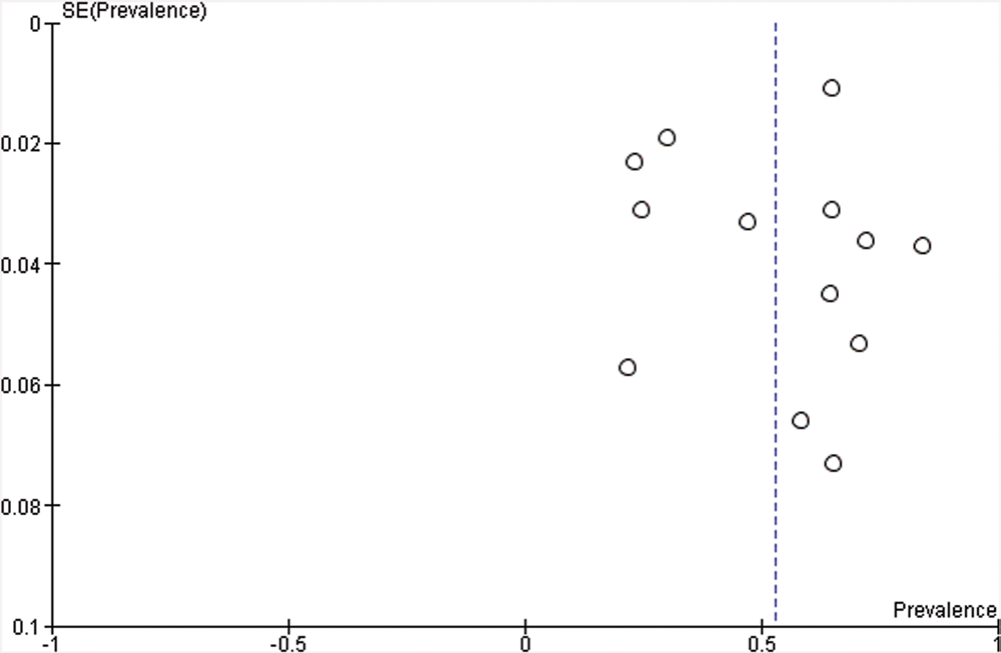
Funnel plot of included studies.

In terms of study quality, the majority of studies had a medium level quality (10/13). The cross-sectional studies failed to make evaluators masked to other aspects of the status of the participants. Quality assurance, methods to handle missing data and expected follow-up were absent in these cross-sectional studies as well. As for prospective cohort study, 2 of the 5 studies demonstrated high quality in which sample came from a national clinical registry study in China and 3 rural villages in the US. The most failed criteria for the rest of the prospective cohort studies were lack of ascertainment of stroke, statement of no history of poor sleep quality and acceptable length of time of follow-up. Detailed information is present in Tables 2 and 3. Funnel plot (Fig. 2) did not detect publication bias.

**Table 2:** Quality of evidence of cross-sectional studies (n = 8).

Study	Item 1	Item 2	Item 3	Item 4	Item 5	Item 6	Item 7	Item 8	Item 9	Item 10	Item 11	Total score
Ho et al,^[12]^ Hongkong	Yes	Yes	Yes	Yes	Unclear	Unclear	Yes	Yes	Unclear	Yes	No	6
Khazaei et al,^[13]^ Iran	Yes	Yes	Yes	Yes	Unclear	Unclear	Yes	Yes	Unclear	Yes	No	6
Sonmez et al,^[15]^ Cyprus	Yes	Yes	Yes	Yes	Unclear	Unclear	Yes	Yes	Unclear	Yes	No	6
Pereira et al,^[16]^ Brazil	Yes	Yes	Yes	Yes	Unclear	Unclear	Yes	Yes	Unclear	Yes	No	6
Kasai et al,^[18]^ Japan	Yes	Yes	Yes	Yes	Unclear	Unclear	Yes	Yes	Unclear	Yes	No	6
Zhao et al,^[20]^ China	Yes	Yes	Yes	Yes	Unclear	Unclear	Yes	Yes	Unclear	Yes	No	6
Nguyen et al,^[24]^ Vietnam	Yes	Yes	Yes	Yes	Unclear	Unclear	Yes	Yes	Unclear	Yes	No	6
de Oliveira et al,^[23]^ Brazil	Yes	Yes	Yes	Yes	Unclear	Unclear	Yes	Yes	Unclear	Yes	No	6

Item 1: Define the source of information (survey, record review); Item 2: List inclusion and exclusion criteria for exposed and unexposed subjects (cases and controls) or refer to previous publications; Item 3: Indicate time period used for identifying patients; Item 4: Indicate whether or not subjects were consecutive if not population-based; Item 5: Indicate if evaluators of subjective components of study were masked to other aspects of the status of the participants; Item 6: Describe any assessments undertaken for quality assurance purposes (e.g., test/retest of primary outcome measurements); Item 7: Explain any patient exclusions from analysis; Item 8: Describe how confounding was assessed and/or controlled; Item 9: If applicable, explain how missing data were handled in the analysis; Item 10: Summarize patient response rates and completeness of data collection; Item 11: Clarify what follow-up, if any, was expected and the percentage of patients for which incomplete data or follow-up was obtained.

**Table 3:** Quality of evidence of prospective cohort studies (n = 5).

Study	Selection				Comparability	Outcome			Total score
	Item 1	Item 2	Item 3	Item 4	Item 5	Item 6	Item 7	Item 8	
Zhang et al,^[14]^ China	1	1	0	0	2	1	0	1	6
He et al,^[19]^ China	1	1	0	0	2	1	0	1	6
Fan et al,^[21]^ China	1	1	0	1	2	1	1	1	8
Xiao et al,^[17]^ China	1	1	0	0	2	1	0	1	6
Brutto et al,^[22]^ USA	1	1	0	1	2	1	1	1	8

Item 1: Exposed cohort representativeness; Item 2: Non-exposed cohort selection; Item 3: Exposure Ascertainment; Item 4: Absence of outcome at baseline; Item 5: Comparability of cohorts; Item 6: Outcome Ascertainment; Item 7: Long enough follow up; Item 8: Follow up adequacy.

### 3.3. Prevalence of poor sleep quality

**Figure 3.**
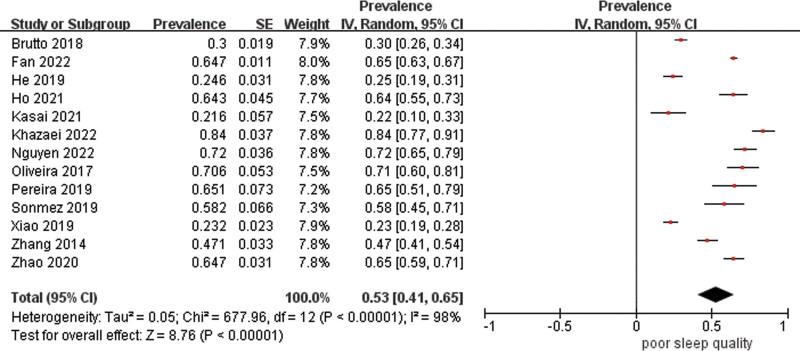
Random effects meta-analysis of all included studies.

**Figure 4.**
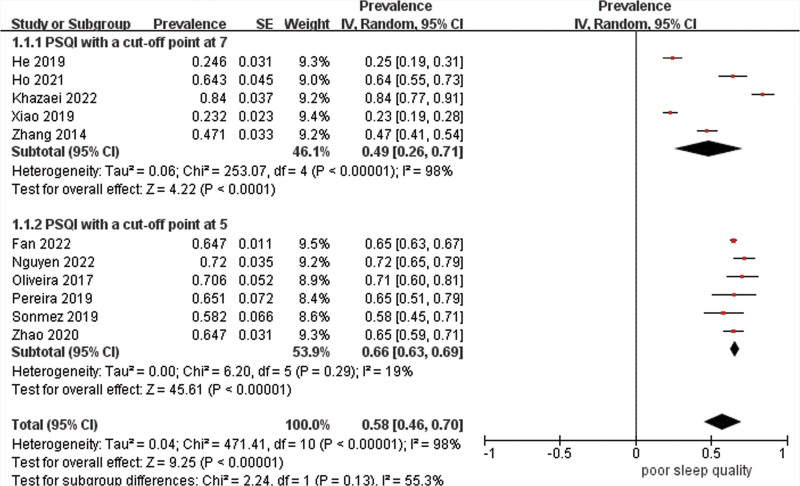
Random effects meta-analysis of studies that used PSQI. PSQI = Pittsburgh Sleep Quality Index.

**Figure 5.**
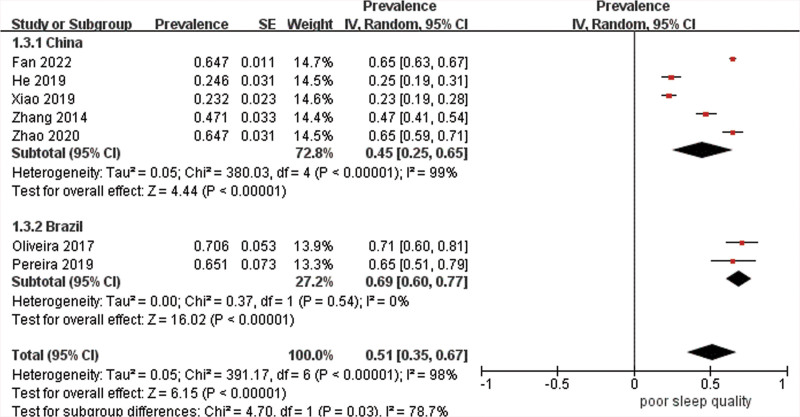
Random effects meta-analysis of studies in different countries.

**Figure 6.**
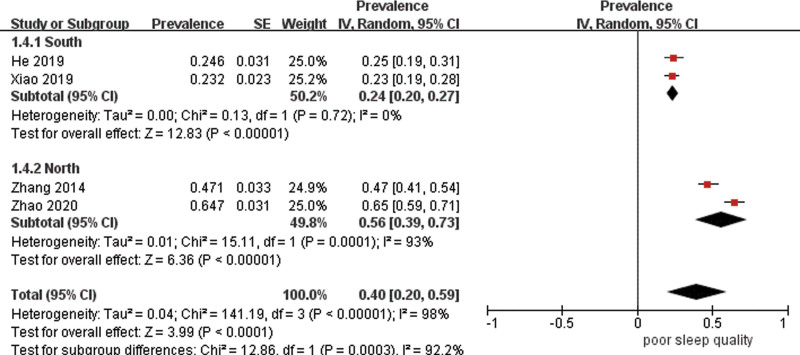
Random effects meta-analysis of studies in different regions in China.

**Figure 7.**
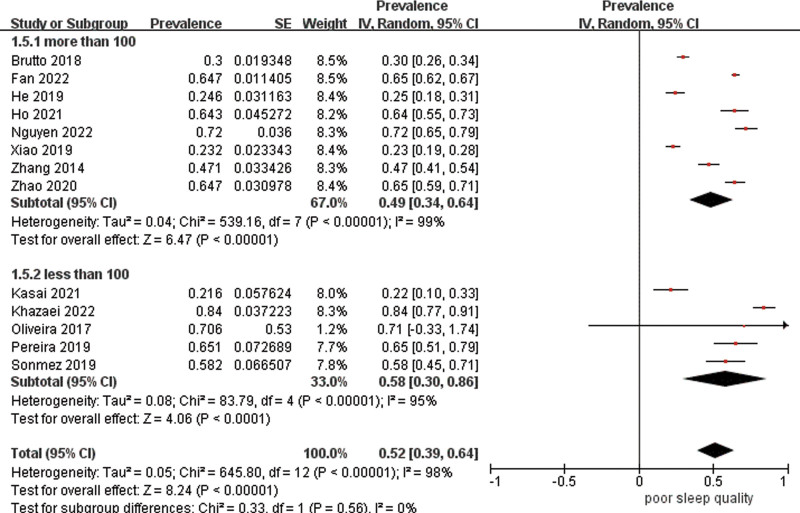
Random effects meta-analysis of studies with different sample size.

Data were pooled for 13 studies (n = 3886) to estimate the prevalence of poor sleep quality with the result of 53% (95% CI 41–65%) with high heterogeneity (*I*^2^ = 98%) (Fig. 3). As estimates varied across studies, stratified analyses were carried out. The combined prevalence estimate of studies using PSQI was 58% (95% CI 46–70%, *I*^2^ = 98%). Subgroups with a cutoff point of 7 had a prevalence of 49% (95% CI 26–71%, *I*^2^ = 98%), whereas those with a cutoff point of 5 had a higher prevalence of 66% (95% CI 63–69%, *I*^2^ = 19%), but the difference was not significant (χ^2^ = 2.24, df = 1, *P* = .13) (Fig. 4). Additionally, studies that were conducted in China had a significantly lower prevalence estimate of 45% (95% CI 25–65%) than those carried out in Brazil of 69% (95% CI 60–77%) (*P* = .03), though there was high heterogeneity between studies in China (Fig. 5). Studies from other countries were not sufficient to be included in the meta-analysis. Further analysis showed that participants from northern regions of China revealed a significantly more than 2-fold higher estimate (56%, 95% CI 30–73%) than those from southern regions of China (24%, 95% CI 20–27%) (*P* < .001) (Fig. 6). Based on methodological factors, there was little to no evidence of a difference between prevalence estimate in studies with sample size of 100 or more (49%, 95% CI 34–64%) and those with less than that number (58%, 95% CI 30–86%) (*P* = .56) (Fig. 7). Other criteria such as time of assessment and study design showed no evidence of a difference between prevalence of poor sleep quality in studies as well.

## 4. Discussion

This systematic review demonstrates that more than half of patients with stroke complain from poor sleep quality, though this estimate should be treated with caution due to high heterogeneity. Geographical location could explain the prevalence variation between studies.

Due to the difference in cutoff point, we made subgroup analyses, but the results failed to explain the prevalence variation between studies, though there is no heterogeneity in the studies using PSQI with a cutoff point at 5 (*I*^2^ = 19%). In a previous review, PSQI was regarded as the most commonly used tool to measure subjective self-report sleep quality and quantify sleep quality with sound reliability and validity.^[25]^ A cutoff score of 5 was proposed in the original paper to distinguish poor and good quality sleepers.^[9]^ However, in order to balance sensitivity and specificity in ROC curve analysis among different populations, previous studies also proposed different cutoff score such as 6 and 7.^[25]^ This reflected the clinical use of PSQI globally. However, existing evidence did not show the variation of prevalence of poor sleep quality attributed to different cutoff point.

Another factor that we considered in subgroup analysis was geographical region. Compared with other regions, we identified significantly lower prevalence of poor sleep quality in Asian population. This may be caused by different epidemiological patterns of stroke in different regions. It has been shown that younger age and less likelihood of low physical activity were the characteristics of stroke in Asia,^[26,27]^ which may be related to lower prevalence of poor sleep quality. This was consistent with a previous review regarding the association between physical activity and quality of sleep.^[28]^ China suffers great burden of stroke. Since most of the included studies were from China, we stratified the location within the country and found a significant difference in the prevalence of poor sleep quality following stroke between northern and southern part of China. Another meta-analysis of prevalence of fatigue after stroke attributed similar difference to psychosocial factors.^[29]^ More evidence is needed to confirm such difference and test the reasons in the future.

We further investigated methodological factors in the subgroup analyses. Despite that the range t of prevalence estimate of poor sleep quality in studies with sample size over 100 was narrower than those with less than 100, there is no evidence of a significant difference between the 2 groups. This could be explained by the fact that the condition of sleep quality is unlike rare diseases that require larger sample size to identify.^[30]^ We did not find time of assessment and study design explained the poor sleep quality prevalence variation between studies, which was inconsistent with the results from another population.^[31]^ This requires further research to explore the impact of methodology on identifying the condition.

There are 2 main limitations in this study. Firstly, due to the insufficient number of studies or difficulty in retrieving data, some criteria were not dissected in the subgroup analyses, such as age and stroke type. Secondly, it would be informative to compute the pooled proportion of participants in each PSQI category but data were lacking in most studies.

## 5. Conclusion

This is the first systematic review to report pooled prevalence estimates of poor sleep quality. Our study demonstrates that poor quality of sleep is prevalent among stroke survivors. This condition varies in terms of occurrence between studies, but geographical location might partially explain this variation. Other substantial between-study variability in prevalence was not easily explained by differences in either methodology or participant characteristics. Therefore, future efforts are needed to identify the factors contributing to it, investigate the mechanisms that lead to poor sleep quality following stroke and develop effective intervention strategies to tackle this issue.

## Acknowledgments

We would like to thank our colleagues for providing language help and encouragement during paper revision.

## Author contributions

**Data curation:** Ye Luo, Guofeng Yu.

**Funding acquisition:** Ye Luo, Guofeng Yu.

**Methodology:** Yuanfei Liu.

**Project administration:** Yinge Zhu.

**Resources:** Chengjun Zhuge.

**Software:** Yuanfei Liu.

**Writing – original draft:** Ye Luo, Yuanfei Liu.
